# Planetary Health Diet and Body Mass Distribution in Relation to Kidney Health: Evidence from NHANES 2003–2018

**DOI:** 10.3390/nu17162692

**Published:** 2025-08-20

**Authors:** Guido Gembillo, Luca Soraci, Maria Elsa Gambuzza, Maria Princiotto, Antonino Catalano, Edlin Villalta, Salvatore Silipigni, Giada Ida Greco, Andrea Corsonello, Domenico Santoro

**Affiliations:** 1 Unit of Nephrology and Dialysis, Department of Clinical and Experimental Medicine, University of Messina, 98125 Messina, Italy; dsantoro@unime.it; 2 Unit of Geriatric Medicine, Italian National Research Center on Aging (IRCCS INRCA), 87100 Cosenza, Italy; g.greco@inrca.it (G.I.G.); a.corsonello@inrca.it (A.C.); 3 Territorial Office of Messina, Ministry of Health, 98100 Messina, Italy; me.gambuzza@sanita.it; 4 Centre for Biostatistics and Applied Geriatric Clinical Epidemiology, Italian National Research Center on Aging (IRCCS INRCA), 87100 Cosenza, Italy; m.princiotto@inrca.it; 5 Unit and School of Geriatrics, Department of Clinical and Experimental Medicine, University Hospital of Messina, 98125 Messina, Italy; catalanoa@unime.it; 6 Independent Researcher, 87100 Cosenza, Italy; edlinvillalta@gmail.com; 7 Department of Biomedical Sciences and Morphologic and Functional Imaging, Policlinico “G. Martino”, University of Messina, 98125 Messina, Italy; salvo.sili@gmail.com; 8 Department of Pharmacy, Health and Nutritional Science, University of Calabria, 87036 Rende, Italy

**Keywords:** CKD, DKD, diabetic nephropathy, older, PHDI, planetary health diet

## Abstract

**Background/Objectives:** Chronic kidney disease (CKD) and diabetic kidney disease (DKD) are growing public health challenges. While diet and body composition influence metabolic and renal health, their combined role remains underexplored. This study investigates the association between the Planetary Health Diet Index (PHDI), body mass distribution, and the prevalence of CKD and DKD in U.S. adults. **Methods**: We analyzed data from 8093 adults aged ≥40 years from NHANES 2003–2018. PHDI was computed using two 24 h dietary recalls. Body composition was assessed using dual-energy X-ray absorptiometry (DXA), focusing on the android-to-gynoid fat ratio (AGFR) and lean mass ratio (AGLR). Survey-weighted linear and logistic regressions evaluated cross-sectional associations between PHDI score, body composition indices, and prevalence of CKD and DKD. Mediation analyses explored AGLR, AGFR, and body mass index (BMI) as potential mediators of the association between PHDI score and either CKD or DKD. **Results**: Higher PHDI scores were mildly associated with lower odds of CKD (OR per 10-point increase: 0.91; 95% CI: 0.83–0.99) and DKD (OR: 0.86; 95% CI: 0.76–0.97). Greater PHDI scores correlated with lower BMI, AGFR, and AGLR. Among participants with diabetes, AGLR mediated 17% of the relationship between a 10-point increase in PHDI score and decreased DKD prevalence, suggesting central lean mass distribution as a relevant pathway. No significant mediation was observed for AGFR, BMI, or for CKD. **Conclusions:** Adherence to PHD is associated with healthier body composition and lower prevalence of CKD and DKD. These findings support the promotion of dietary strategies that enhance metabolic and renal health in middle-aged and older individuals.

## 1. Introduction

Chronic kidney disease (CKD) represents a global public health concern, affecting over 10–14% of the adult and older population, and contributing to increased morbidity, mortality, and healthcare costs [1]. A major subset of CKD is diabetic kidney disease (DKD), which develops as a microvascular complication in approximately one-third of individuals with long-standing diabetes mellitus and is associated with albuminuria, progressive decline of glomerular filtration rate (GFR), podocyte dysfunction, and renal fibrosis [2].

The pathogenesis of CKD and DKD is multifactorial, involving a complex interplay of metabolic, hemodynamic, and inflammatory mechanisms. Chronic hyperglycemia is a central driver in DKD, inducing non-enzymatic glycation of proteins and lipids, formation of advanced glycation end-products, and increased polyol pathway activity, all of which contribute to glomerular and tubular injury [3,4]. Subclinical systemic and adipose tissue inflammation, mediated by elevated levels of pro-inflammatory cytokines such as TNF-α, IL-1, and IL-6, promotes endothelial dysfunction and tubulointerstitial fibrosis in both CKD and DKD [5,6]; growing evidence suggests that chronic inflammation of adipose tissue may favor both the development of pancreatic β-cell dysfunction as well as glomerular and tubulointerstitial pathology [7,8]. Both obesity and insulin resistance are increasingly recognized as key contributors to CKD pathogenesis through the secretion of adipokines such as leptin, resistin, and visfatin, which induce oxidative stress and mesangial expansion [9]. Oxidative stress, primarily resulting from mitochondrial dysfunction, hyperglycemia, and NADPH oxidase activation, further exacerbates renal injury by damaging cellular structures and amplifying inflammatory signaling [10], thus contributing to the development and progression of DKD and CKD [11].

Among potentially modifiable risk factors for CKD and DKD development and progression, diet emerged as one of the most powerful. Unhealthy dietary patterns, characterized by high intakes of sodium, added sugars, saturated fats, red and processed meats, and ultra-processed foods, are associated with increased risk of CKD, faster decline in kidney function, and increased progression to end-stage renal disease [12,13]; these diets promote metabolic disturbances such as hypertension, dyslipidemia, and insulin resistance and contribute to pro-inflammatory and pro-oxidative milieu that accelerates renal injury [14]. In contrast, plant-centered dietary patterns rich in dietary fiber, unsaturated fats, antioxidants, and phytochemicals have demonstrated protective effects on renal outcomes by modulating systemic inflammation, improving endothelial function, and promoting better glycemic and blood pressure control [13,15,16].

Within this context, the Planetary Health Diet (PHD), recently proposed by the EAT-Lancet Commission, offers a comprehensive and sustainable framework that prioritizes both individual and planetary well-being [17,18]. The PHD emphasizes a predominantly plant-based diet and mostly minimally processed foods, including a generous intake of whole grains, fruits, vegetables, legumes, nuts, and unsaturated oils, while recommending limited consumption of red meat, highly processed foods, added sugars, and refined starches. After PHD conceptualization, Cacau et al. proposed the development and validation of the PHD Index (PHDI) to quantify the adherence to PHD [19]. Recent large-scale cohort studies have demonstrated that higher PHDI scores are associated with favorable health outcomes, including decreased incidence of obesity, metabolic syndrome, cardiovascular disease, and all-cause mortality [19,20,21,22]. PHD proposed an animal/plant protein ratio of approximately 30:70, aligning with the worldwide healthy protein transition plans; plant-based diets have previously shown to mitigate inflammation in CKD [23]. Furthermore, a higher adherence to PHD has been associated with decreased prevalence of sarcopenia and a lower all-cause mortality in patients with CKD. However, the associations between adherence to this diet and the prevalence of chronic renal diseases such as CKD and DKD remained largely unexplored. Given the rising burden of these conditions globally, clarifying this relationship could add insights for public health nutrition strategies.

In parallel, emerging research highlights the importance of body fat distribution and not just total adiposity as a key determinant of renal and metabolic risk [24,25,26]. Conventional anthropometric measures such as body mass index (BMI) are limited in their ability to distinguish between visceral and subcutaneous fat, or to reflect lean tissue mass. Conversely, imaging-based indices like the android-to-gynoid fat mass ratio (AGFR) and android-to-gynoid lean mass ratio (AGLR), derived from dual-energy X-ray absorptiometry (DXA), provide more nuanced assessments of body composition. Higher AGFR reflects greater central (visceral) fat accumulation, which is metabolically active and pro-inflammatory, while elevated AGLR may suggest central lean mass predominance, potentially associated with ectopic fat infiltration and myosteatosis. Increase in AGFR has recently been linked to adverse outcomes, including insulin resistance, metabolic syndrome, and chronic inflammation [27], all of which may predispose individuals to renal injury [28]. Similarly, increased AGLR has been associated with all-cause and cardiovascular mortality in middle-aged and older adults [29] and with overall mortality in individuals with diabetes [30]. Given the convergence of dietary patterns, changes in body mass distribution, and inflammatory stress in the pathophysiology of CKD and DKD, we hypothesized that higher adherence to the PHD would be associated with more favorable body composition and lower kidney disease prevalence. Specifically, this study aimed to explore the cross-sectional associations between PHDI, AGFR, AGLR, and the prevalence of CKD and DKD in a nationally representative sample of U.S. adults drawn from the National Health and Nutrition Examination Survey (NHANES).

## 2. Materials and Methods

### 2.1. Study Population

**Figure 1:**
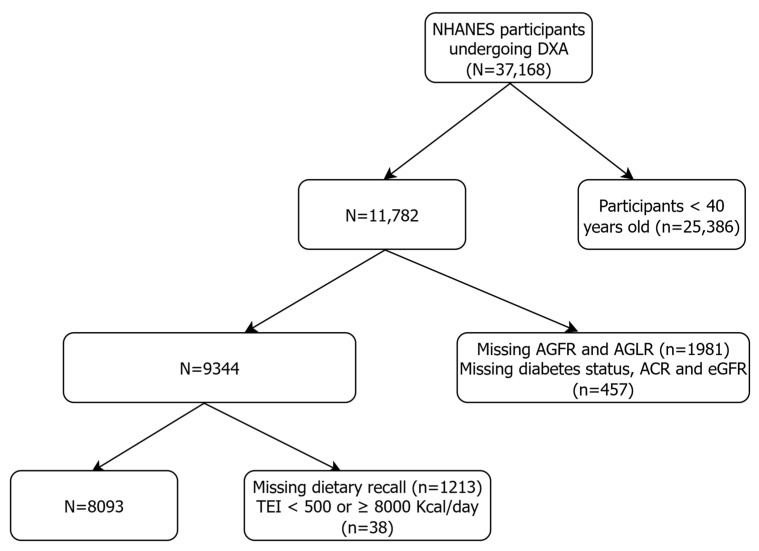
Flowchart of the participant screening process for the analysis.

This study is a cross-sectional analysis based on data from the National Health and Nutrition Examination Survey (NHANES), a continuous, multistage probability survey designed to assess the health and nutritional status of the community-dwelling adult and older US population. A total of 37,168 patients aged 40 years and older and undergoing DXA examination during six wave cycles between 2003 and 2018 were initially included in the present analysis. After excluding individuals younger than 40 years (*n* = 25,386), those with missing android–gynoid ratio measurements (*n* = 1981), and those with incomplete information on diabetes mellitus status, albumin-to-creatinine ratio (ACR), and estimated Glomerular Filtration Rate (eGFR) (*n* = 457), we obtained an intermediate sample of 9344 individuals. Finally, having excluded participants with missing two-day 24 h dietary recall (*n* = 1213) and those with a total energy intake (TEI) <500 or ≥8000 Kcal/day (*n* = 38), we obtained a final sample of 8093 patients to be included in the study. The flowchart of the selection of patients finally included in the study sample is presented in Figure 1.

NHANES protocols are approved by the Ethics Review Board of the National Center for Health Statistics, and all participants provided informed consent. The dataset was fully anonymized and freely accessible at the official NHANES website (https://wwwn.cdc.gov/nchs/nhanes/default.aspx) (accessed on 31 July 2025). Written informed consent was obtained from all participants. The study was conducted following the principles of the Declaration of Helsinki.

### 2.2. Dietary Assessment and PHDI Score Calculation

**Figure 2:**
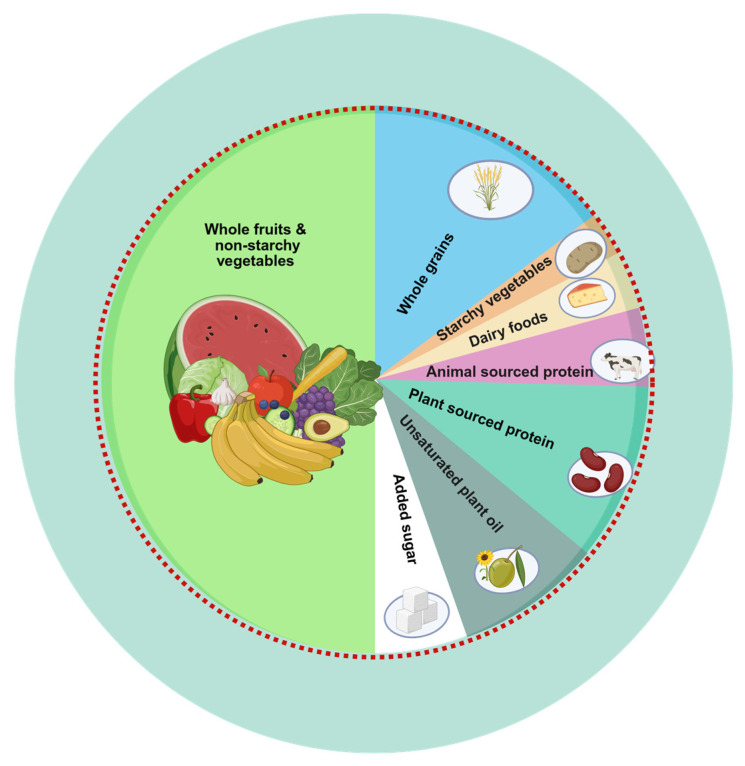
The Planetary Health Diet Plate. A planetary health plate is typically composed of about half fruits and vegetables by volume. The remaining half, in terms of caloric contribution, should mainly include whole grains, plant-based protein sources, and unsaturated plant oils, with the optional addition of small amounts of animal-derived protein. Figure created in BioRender. Soraci, L. (2025) https://BioRender.com/dcia084 and adapted from the EAT-Lancet Commission Summary Report.

Dietary intake was assessed using two non-consecutive 24 h dietary recalls, collected by trained interviewers using the United States Department of Agriculture (USDA) Automated Multiple-Pass Method. Day 1 data were collected in person at the Mobile Examination Center; day 2 data were collected via telephone interviews 3–10 days later, according to methodology presented elsewhere [31]. The average of the two days was used to estimate daily intake of foods and nutrients and total energy intake (TEI) in Kcal/day. Dietary data were then linked to the Food Patterns Equivalents Database, which categorizes foods into the 37 USDA Food Pattern Components, using a food composition table [32]. Food pattern equivalents were then converted into grams/day according to standardized conversion units. Adherence to the PHD was then assessed using the PHDI scoring system proposed by the EAT-Lancet Commission [17] and further adapted to the US population [20] in order to reflect adherence to a nutritionally adequate and environmentally sustainable dietary pattern; the main sources of food included in the PHDI are shown in Figure 2.

More specifically, PHDI includes fourteen components divided into two domains (Table 1):

- Adequacy components represent food groups encouraged for regular consumption due to their nutritional and health-promoting properties. They include whole grains, non-starchy vegetables, whole fruits, legumes (both soy-based and non-soy), nuts, fish and seafood, and unsaturated plant oils (e.g., olive, rapeseed, sunflower, soybean).
- Moderation components represent food groups that should be limited due to their association with adverse health and environmental impacts. These include red and processed meats, dairy products, eggs, saturated fats (e.g., butter, lard, palm oil), starchy vegetables and tubers (e.g., potatoes), poultry, and added sugars or fruit juice.

**Table 1:** PHDI components and criteria for definition of scoring system.

	EAT-Lancet Reference Diet		Scoring Criteria		
	Grams/Day	Kcal/Day	Min Score (0) in g/d	Max Score (10) in g/d	Weighted Score
**Adequacy**					
Whole grains	232 (0–60% of TEI)	811	0	≥75 g/d in M; ≥90 g/d in F	1
Non-starchy vegetables	300 (200–600)	78	0	≥300	1
Whole fruits	200 (100–300)	126	0	≥200	1
Soybean and soy foods	25 (0–50)	112	0	≥50	0.5
Non-soy legumes (e.g dry beans, peas, lentils)	50 (0–100)	172	0	≥100	0.5
Nuts (e.g., peanuts and tree nuts)	50 (0–75)	291	0	≥50	1
Fish and shellfish	28 (0–100)	40	0	≥28	1
Added unsaturated oils (e.g., olive, soybean, rapeseed, peanut oil, sunflower oil)	40 (20–80)	354 (14.16% of TEI)	≤3.5% of TEI	≥21% of TEI	1
**Moderation**					
Tubers and starchy vegetables	50 (0–100)	39	≥200	≤50	1
Dairy	250 (0–500)	153	≥1000	≤250	1
Eggs	13 (0–25)	19	≥120	≤13	1
Red and processed meat (e.g., beef, pork, lamb)	14 (0–28)	30	100	≤14	1
Poultry (e.g., chicken, duck, goose, ostrich)	29 (0–58)	62	≥100	≤29	1
Added saturated fats (e.g., palm oil, coconut oil, dairy fat-butter, margarine, lard, tallow)	11.8 (0–11.8)	96 (3.8% of TEI)	≥10% of TEI	0% of TEI	1
Added sugars and fruit juices	31 (0–31)	120 (4.8% of TEI)	≥25% of TEI	≤5% of TEI	1

Notes: TEI: total energy intake.

Each component was assigned a score ranging from 0 (lowest adherence) to 10 (highest adherence) based on recommended intake ranges. For example, a value of 10 was awarded when component intake met or exceeded the EAT-Lancet recommended minimum (for adequacy components) or stayed below the upper threshold (for moderation components). Partial points were proportionally assigned between minimum and maximum thresholds (Table 1). The points of each of the 14 food components were then summed to obtain an overall PHDI score, ranging from 0 to 150, and representing adherence to the diet, with higher scores indicating greater alignment with the planetary health dietary model.

### 2.3. Diabetes and Kidney Function

Diabetes was defined as either a self-report of diabetes diagnosis by a physician (or another healthcare professional), using items included in the diabetes questionnaires [33], or by the presence of a glycated hemoglobin > 6.5%. Kidney function was assessed using two standard biomarkers: the estimated glomerular filtration rate (eGFR) and the urinary albumin-to-creatinine ratio (ACR). eGFR was calculated by using the race-free CKD-EPI 2021 equation [34], while ACR was determined from spot urine samples by measuring urinary albumin and creatinine concentrations. 

### 2.4. Study Outcomes

Study outcomes were represented by CKD and DKD. CKD was defined as the presence of eGFR < 60 ml/min/1.73 m^2^ based on a single measurement; DKD was identified among individuals with diabetes as having eGFR < 60 ml/min/1.73 m^2^, and/or ACR ≥ 30 mg/g. 

### 2.5. Body Composition Measures

Body composition data were obtained from whole-body DXA scans, conducted using the Hologic QDR 4500A densitometer [35]. Trained technicians followed standardized protocols, and scans were processed using Hologic APEX software version 3.0 for the 2003–2004 cycle and v 3.2 for the 2011-2018 cyclesThe android and gynoid regions were automatically defined based on anatomical landmarks: the android region as the area round the abdomen and visceral cavity, and the gynoid region corresponding to the hip and thigh area below the waistline. AGLR and AGFR were then calculated by dividing android and gynoid lean and fat mass components respectively.

### 2.6. Study Covariates

Demographic variables included in the analysis were age, gender, and race/ethnicity; BMI was calculated as weight in kilograms divided by height in meters squared, both measured in mobile examination centers with standard protocols; obesity was defined as having a BMI ≥ 30 Kg/m^2^, according to the WHO definition for international BMI classification. High-density lipoprotein cholesterol (HDL-C) was used as a measure of dyslipidemia in analyses, while glycated hemoglobin (HbA1c %) and duration of diabetes were considered to assess the severity of diabetes mellitus. Comorbidities also included history of heart disease (congestive heart failure or coronary artery disease), hypertension, respiratory disease (chronic bronchitis or emphysema), stroke, and cancer. TEI was also considered in the analysis as a measure of two-day weighted caloric intake; to account for potential misreporting and facilitate interpretation, total energy intake was categorized into three groups: moderate intake (1500–3000 Kcal/day), considered as the reference category in the regression models; low intake (<1500 Kcal/day); and high intake (>3000 Kcal/day). Daily protein intake was also considered and normalized to adjusted body weight (ABW), calculated by using the following equations: ABW = IBW + 0.25 × (Actual Body Weight − IBW), where IBW is the ideal body weight calculated with the Devine formula [36]. Detailed descriptions of blood collection and processing procedures were provided on the NHANES website (https://wwwn.cdc.gov/nchs/nhanes/continuousnhanes/) (accessed on 31 July 2025).

### 2.7. Statistical Analysis

To guarantee that the data in our investigation were nationally representative, we used the weights that the National Center for Health Statistics suggested. Descriptive statistics were first computed for baseline characteristics across PHDI score quintiles. Continuous variables were presented as means and standard deviations (SD) or medians with interquartile ranges (IQR) and compared using survey-weighted linear regression models with Wald test or design-based Kruskal–Wallis rank test, respectively. Categorical variables were summarized as frequencies (%) and compared using the Rao–Scott adjusted chi square test.

To evaluate the baseline correlation between diet adherence and imaging body fat indices, we computed survey-weighted linear regression models evaluating the association between a 10-point increase in PHDI score and either imaging body composition indices (AGLR and AGFR, expressed as percent change) or BMI, adjusting sequentially for demographic, lifestyle, and clinical covariates. To evaluate the association between PHD adherence and CKD/DKD, we conducted survey-weighted logistic regression models using PHDI score as both a continuous variable (as a 10-point increase in PHDI score) and PHDI score quintiles, with the lowest quintile (lowest PHD adherence) set as the reference level. Three regression models were built as follows: model A, adjusted for age, sex, and race; model B, further adjusted for hypertension, heart disease, respiratory disease, cancer, stroke, diabetes (only when the outcome was CKD), HDL, and TEI; model C, equal to Model B + obesity and AGLR tertiles; and model D, equal to Model B + obesity and AGFR tertiles. Finally, to explore potential mechanistic pathways linking PHDI score and kidney outcomes, we performed mediation analysis with bootstrap resampling with 1000 iterations and adjusted for relevant covariates to determine whether AGLR%, AGFR%, and BMI mediated the relationship between a 10-point increase in PHDI score and CKD/DKD prevalence. The model followed a standard path analysis approach: Path A: the effect of a 10-point increase in PHDI score on the mediator (e.g., AGFR%). Path B: the effect of the mediator on the kidney outcome, adjusting for a 10-point change in PHDI score. Path C: the total effect of a 10-point increase in PHDI score on the outcome. Path C′: the direct effect of a 10-point increase in PHDI score on the outcome after including the mediator. The indirect effect was derived from the product of paths A and B (*A × B*). The proportion mediated was calculated as the ratio of the indirect effect to the total effect, expressed as (indirect effect/[indirect effect + direct effect]) × 100%. All effects were expressed as regression coefficients, and statistical significance was determined using bias-corrected bootstrap confidence intervals. All statistical analyses were performed using R software (version 4.6, R Foundation for Statistical Computing, Vienna, Austria) incorporating the mediation and survey packages for mediation modeling, and complex survey design adjustments, respectively.

## 3. Results

### 3.1. Baseline Characteristics of the Study Population

The baseline characteristics of study participants are reported in Table 2. Of the 8093 participants, 52% were female, predominately white race (72.6%), and a mean age of 52 years; among them, approximately 39% were categorized as obese, 11.5% were diagnosed with diabetes (of which 29% were classified as DKD), and 4% had a CKD diagnosis. The median PHDI score was 73.8, with over 60% of the study population being characterized by a medium caloric intake (TEI of 1500–3000 Kcal/day) and a median protein intake of 1.2 g/Kcal/day.

Demographic, metabolic, and clinical characteristics of the study population across PHDI score quintiles are reported in Supplementary Table S1. Increasing PHDI score quintiles represented progressively increasing adherence to PHD and were characterized by a slightly higher age, and a higher prevalence of women; furthermore, higher PHDI score quintiles presented significantly lower prevalences of obesity, hypertension, respiratory disease, and stroke, as well as lower median (IQR) values of imaging body fat parameters (AGLR, and AGFR) and BMI; although the prevalence of diabetes did not differ across PHDI score quintiles, patients belonging to the highest quintile had slightly lower median HbA1c % values and a slightly lower daily caloric and protein intake. Finally, only the highest PHDI score quintile, representing patients with the highest adherence to the PHD, was associated with a significantly lower prevalence of both CKD and DKD.

### 3.2. Association Between PHDI Score, BMI, and Imaging Body Fat Indices (AGLR and AGFR)

Multivariate survey-weighted linear regression models showing the association between continuous PHDI score (expressed as change in 10-point PHDI score), AGLR, AGFR, and BMI are reported in Table 3.

An increasing PHDI score has been consistently associated with lower AGLR%, AGFR%, and BMI, even after adjusting for multiple covariates; more specifically, a 10-point increase in the PHDI score was associated with a 0.5% decrease in AGLR, a 0.7% decrease in AGFR, and a 0.3-point decrease in BMI. Among study covariates, both age, white race, and female gender influenced AGLR, AGFR, and BMI; indeed, increasing age was associated with increasing AGLR and AGFR values, as well as decreasing BMI; female gender was associated with a slight decrease in AGLR values and a very strong decrease in AGFR (median 15% decrease vs. male gender), and a slight BMI increase. Among other covariates associated with the indices, the presence of diabetes or hypertension was associated with an increase in both AGLR, AGFR, and BMI; heart disease and respiratory disease were associated with increased AGLR but did not affect other indices.

### 3.3. Association Between PHDI and the Prevalence of CKD

The association between continuous PHDI score and PHDI score quintiles with CKD in the whole study population was investigated by using survey-weighted logistic regression models, with OR (95%CI) reported in Table 4 and Supplementary Table S2, respectively. Across all models, higher adherence to the PHD was significantly associated with lower odds of CKD: indeed, in continuous analyses (Table 4), each 10-point increase in PHDI score was associated with a 9–13% lower prevalence of CKD; in all models, older age and presence of diabetes, hypertension, heart disease, and stroke were independently associated with higher CKD prevalence, while white race was consistently associated with lower odds of CKD. Total energy intake below 1500 kcal/day was also associated with significantly higher CKD prevalence across models. Conversely, AGLR, AGFR, and obesity were not significantly associated with CKD.

However, increasing PHDI score quintiles showed a non-significant trend of association with decreased CKD prevalence (Supplementary Table S2).

### 3.4. Association Between PHDI and the Prevalence of DKD

Among the 930 individuals with diabetes in the NHANES cohort, survey-weighted logistic regression models revealed that higher adherence to the PHD was significantly associated with reduced odds of DKD (Table 5 and Supplementary Table S3). Moreover, each 10-point increase in PHDI was associated with a 13–15% reduction in DKD prevalence across all models. Other covariates independently associated with higher DKD prevalence included older age, hypertension, heart disease, and low energy intake (<1500 kcal/day), which consistently showed ORs > 1.5 across models. Notably, white race and female gender were significantly associated with lower odds of DKD in all adjusted models. The protective effect of female gender was strongest in Model C (OR = 0.49; 95% CI: 0.30–0.79; *p* < 0.01).

Moreover, body composition appeared to influence DKD prevalence mainly through AGLR. In Model C, participants in the highest tertile of AGLR (indicating greater central lean mass distribution) had more than twice the odds of DKD compared to those in the lowest tertile (OR = 2.40; 95% CI: 1.39–4.16; *p* < 0.01). These results were confirmed in analyses including PHDI quintiles instead of continuous index, with the highest quintile being associated with decreased odds of DKD (Supplementary Table S3).

### 3.5. Mediation Analysis of the Associations Between PHDI, DKD, and CKD by AGLR and BMI

**Figure 3:**
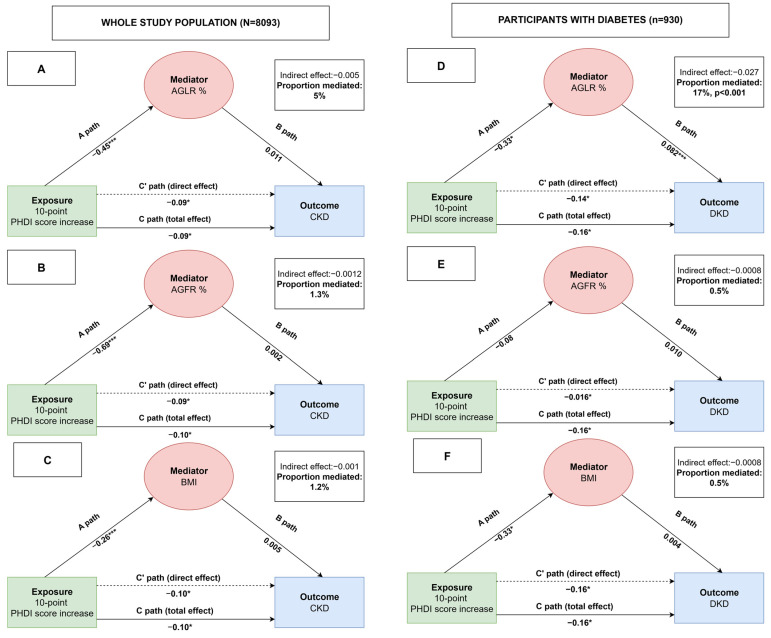
Mediation analyses showing the associations between PHDI, mediators (AGLR, AGFR, and BMI) and study outcomes. A mediation model illustrating the relationship between the independent variable represented by a 10-point increase PHDI score (X) and the dependent variables CKD or DKD (Y) through mediators (M) AGLR (panels **A** and **D**), AGFR (panels **B** and **E**), and BMI (panels **C** and **F**). Path A represents the effect of X on M, path B represents the effect of M on Y, and path C′ (C-prime) represents the direct effect of X on Y after accounting for the mediator. Path C represents the total effect of X on Y (i.e., before including the mediator). The indirect effect of X on Y through M is calculated as the product of paths A and B (*A × B*). The proportion mediated is the indirect effect (*A × B*) divided by the total effect (C). *** *p* value < 0.001; * *p* value < 0.05.

In the mediating analysis, we investigated whether AGLR, AGFR, and BMI may mediate the relationship between PHDI and either CKD or DKD (Figure 3).

More in detail, a 10-point increase in PHDI score was associated with a decreased prevalence of CKD and DKD; among body mass indices, increased AGLR % was associated with an increased prevalence of DKD; mediation analysis showed that about 17% of the total effect of PHDI on DKD was explained by a reduction in AGLR%, indicating that a better central lean mass distribution may contribute meaningfully to the observed protective association.

No significant mediation was observed for AGFR, BMI, or for chronic kidney disease (CKD) in the overall population.

## 4. Discussion

This study examined the association between PHDI, a measure of alignment with a sustainable, plant-centered dietary pattern, the prevalence of CKD and DKD, and the mediating role played by AGLR, AGFR, and BMI in a representative U.S. adult population. Our findings show that higher PHDI was independently associated with lower prevalence of both CKD and DKD. Furthermore, higher diet adherence was associated with improvement in all body mass indices, i.e., a decrease in AGLR, AGFR, and BMI values. Importantly, the relationship between PHDI and DKD was partially mediated by the AGLR, with 17% of the total effect explained by differences in lean mass distribution. No significant mediation was found for AGFR or BMI, nor was mediation observed for CKD.

To our knowledge, this is the first study to investigate the association between PHDI and the prevalence of CKD and DKD in community-dwelling adults and to evaluate the potential mediating role of body fat indices. Previous cohort studies had shown that plant-based and Mediterranean-style diets other than PHD are associated with improved kidney health and cardiometabolic profiles [37,38]; however, the role of PHD on renal health remains insufficiently explored; the PHDI operationalized the EAT-Lancet Commission’s dietary recommendations by quantifying adherence to a dietary model that prioritizes human health while minimizing environmental impact [17,18]. This framework emphasizes the consumption of whole grains, legumes, fruits, vegetables, nuts, and unsaturated oils, while recommending a reduction in the intake of red and processed meats, added sugars, and high-fat dairy. These food groups are rich in fiber, antioxidants, and unsaturated fatty acids, all of which are associated with anti-inflammatory and vasculoprotective effects [39,40]. A recent study using a similar NHANES cohort has demonstrated that PHDI has been associated with a decreased prevalence of obesity, better cardiometabolic profile, and lower all-cause mortality [39]. Building on prior evidence, our study adds novel findings on the cross-sectional associations between PHDI and prevalence of CKD and DKD, and the potential intermediary role of lean mass distribution

Several biological pathways might plausibly explain the observed associations, although these remain speculative and require confirmation in longitudinal or interventional studies. First, a higher intake of plant-based foods, characterized by a low glycemic index and a high content in phytochemicals, may improve glycemic control and lipid profiles, thus potentially reducing glomerular hyperfiltration and slowing nephron injury in DKD [41,42]; this hypothesis is partly confirmed by our results, as patients with higher PHDI were characterized by a better metabolic and cardiovascular profile (e.g., lower glycated hemoglobin and body fat indices, higher serum HDL), along with a lower prevalence of both CKD and DKD.

Second, PHD contains many anti-inflammatory and antioxidant nutrients such as fruits, vegetables, whole grains, legumes, and nuts, which provide vitamin C, E, carotenoids, and flavonoids; such compounds may lower systemic inflammation and oxidative stress, recognized as key contributors to endothelial dysfunction and podocyte damage, both implicated in CKD and DKD pathogenesis [43,44]. In contrast, PHD advises limiting ultra-processed and animal-based foods, including red and processed meats, added sugars, and saturated fats, which have been associated with multiple harmful effects [45,46,47,48]. These include elevated renal acid load, increased uremic toxins, promotion of insulin resistance, and elevated levels of pro-inflammatory cytokines such as TNF-α and IL-6. Moreover, lower saturated fat intake combined with greater consumption of dietary fiber may help improve gut dysbiosis and systemic metabolic dysfunction, processes that can further accelerate DKD progression [49].

Third, PHD is characterized by a lower dietary acid load, which stems from higher intakes of potassium, magnesium, and bicarbonate precursors and lower intakes of sulfur-containing amino acids abundant in animal proteins. High acid load has been linked to increased renal ammoniagenesis, tubulointerstitial fibrosis, and impaired bicarbonate reabsorption, all of which may exacerbate kidney injury. Conversely, alkalinizing diets may help preserve nephron function and reduce tubular stress, also decreasing the risk of metabolic acidosis, a severe complication of CKD [50]. Furthermore, the lower intake of salts due to a decreased consumption of highly processed food is associated with decreased systemic blood and glomerular pressure and improved proteinuria and natriuresis, all of which independently accelerate CKD and DKD progression [51]. However, while these pathways are biologically plausible and consistent with prior literature, they cannot be confirmed by our cross-sectional study results and future longitudinal and mechanistic research is required to establish their temporal sequence and causal relevance.

The second most relevant finding of our study was the inverse association between PHDI and DXA-derived body fat metrics, including AGLR and AGFR. Previous studies reported that AGLR, which reflects the relative distribution of lean mass, has been associated with DKD and diabetic retinopathy in diabetic patients of the NHANES cohort [24,25] as well as increased all-cause and cause-specific mortality [29]. Our finding that 15% of the protective effect of PHDI on DKD is mediated through AGLR suggests that diet may influence renal health via its impact on muscle quality and distribution, possibly by modulating ectopic fat infiltration in lean tissue or improving skeletal muscle insulin sensitivity. The absence of significant mediation by BMI or AGFR underscores that lean mass quality and distribution, rather than overall adiposity or fat location, may play a more prominent role in the association with DKD. This highlights the limitations of conventional obesity metrics when evaluating diet-related disease mechanisms, especially in metabolically complex conditions like diabetes. Conversely, emerging research suggests quality and distribution of lean mass are critical determinants of metabolic and renal health; myosteatosis, the pathological fat infiltration into skeletal muscle, may contribute to insulin resistance, impaired mitochondrial function, chronic inflammation, and sarcopenic obesity [52,53]; these consequences can lead to an increased release of pro-inflammatory cytokines which can affect renal podocytes via systemic and paracrine signaling pathways [54]. Furthermore, increased lean mass in the android region, especially in the presence of systemic insulin resistance and fat infiltration, may further impose greater hemodynamic and metabolic load on central organs. Indeed, given the proximity of the android compartment to visceral adipose tissue and abdominal organs, this muscle mass may be more prone to dysfunctional remodeling and could amplify cardiorenal stress, particularly affecting renal perfusion and vascular resistance [55,56]; thus, a high AGLR may reflect a regionally unbalanced and metabolically compromised lean mass profile that promotes systemic inflammation and renal injury. PHD adherence may help mitigate these changes by improving mitochondrial function, reducing oxidative stress, and enhancing muscle insulin sensitivity.

This study has several notable strengths. It utilizes a large, nationally representative sample of U.S. adults with high-quality clinical and dietary data and incorporates DXA–derived body composition measurements to assess lean and fat mass distribution with greater precision than standard anthropometrics. However, since DXA is not as effective in distinguishing intramuscular fat infiltration as computed tomography or magnetic resonance imaging, further studies are needed to confirm these findings. Second, the application of the PHDI offers a contemporary framework to evaluate diet quality in the context of both human and environmental health. Furthermore, the use of mediation analysis provides novel insight into potential biological pathways linking diet and diabetic kidney disease.

However, many important limitations must also be acknowledged. First, this is a cross-sectional study assessing dietary patterns and kidney measures within the same examination cycle. The results therefore reflect the prevalence rather than incidence of CKD and DKD, thus precluding causal inference and limiting the ability to assess changes over time. Second, the cross-sectional study design implies a risk of reverse causation, as individuals with CKD or DKD may have altered their diets after diagnosis, making current dietary intake an imperfect proxy for prior long-term habits biasing the strength and direction of observed associations. Third, dietary intake was self-reported using two-day 24 h recalls, which may misclassify habitual diet due to day-to-day variability and recall bias. Fourth, in this study CKD was defined as reduced eGFR (<60 mL/min/1.73 m^2^) based on a single measurement. Albuminuria was not included in this definition, so individuals with preserved eGFR but elevated UACR were not classified as having CKD. This approach, applied to maintain a clear distinction between CKD and DKD, may result in slightly lower CKD prevalence estimates and should be interpreted as representing a conservative and more functionally defined CKD phenotype. Fifth, DXA data was available only for a subset of participants, potentially affecting generalizability. Additionally, while AGLR served as a proxy for lean mass distribution, direct measures of muscle quality, such as imaging of myosteatosis or muscle function tests, were not included. Sixth, as the study was conducted in a U.S. population with specific dietary habits, body composition characteristics, and healthcare system factors, the findings may not be directly generalizable to populations in other countries with differing cultural or dietary contexts. Furthermore, as most participants had mostly preserved eGFR and ACR values, stratification by CKD stages or ACR categories was not undertaken, as the small numbers in certain subgroups could have limited statistical power and the reliability of estimates. Finally, although the PHDI reflects adherence to a sustainable diet, cultural and individual variability in dietary patterns may limit its universal applicability.

## 5. Conclusions

This study provides the first evidence that greater adherence to a sustainable, plant-centered dietary pattern, as captured by the PHDI, is associated with healthier body composition and a significantly lower prevalence of CKD and DKD in U.S. adults. Among individuals with diabetes, the relationship between PHDI and DKD was partially mediated (19%) by central lean mass distribution (AGLR), suggesting a novel pathway linking dietary quality to renal protection via muscle composition.

These findings reinforce the importance of dietary strategies that emphasize whole, plant-based foods for both kidney and metabolic health. The results also highlight the value of considering lean mass distribution and potential early muscle alterations, such as myosteatosis, as intermediary mechanisms in the diet–kidney axis. DXA-derived metrics like AGLR potentially offer advantages over traditional markers like BMI by providing more detailed insights into regional fat and lean mass distribution. Future research should continue to explore these indices to improve risk stratification and inform targeted interventions.

From a public health perspective, promoting sustainable dietary patterns may offer dual benefits, improving chronic disease outcomes and supporting planetary health goals, in line with emerging global dietary guidelines. Future longitudinal studies with repeated dietary and body composition assessments are needed to confirm these associations and establish temporality.

## Figures and Tables

**Table 2:** Characteristics of the participants included in the analysis.

Characteristics	N = 8093
Age, years, mean (SD)	52.2 (8.2)
Female gender, n (%)	4210 (52.0)
White race, n (%)	5879 (72.6)
Hypertension, n (%)	2874 (35.6)
Glycated hemoglobin, mean (SD)	5.7 (1.0)
Diabetes, n (%)	930 (11.5)
Heart disease, n (%)	333 (4.1)
Respiratory disease, n (%)	629 (7.8)
Stroke, n (%)	171 (2.1)
Cancer, n (%)	735 (9.1)
AGLR, median (IQR)	0.52 (0.48–0.56)
AGFR, median (IQR)	0.55 (0.43–0.70)
BMI, mean (SD)	29.2 (6.2)
Obesity, n (%)	3112 (38.6)
HDL, mg/dl, median (IQR)	52 (42–63)
PHDI score, median (IQR), [range]	73.8 (62.8–83.6) [21.2–127.8]
TEI, Kcal/day, median (IQR)	2101.0 (788.4)
TEI category, Kcal/day, n (%)	
1500–3000	5180 (64.0)
<1500	1878 (23.2)
>3000	1035 (12.8)
Total protein intake/ABW (g/Kg/day), median (IQR)	1.2 (0.9–1.4)
eGFR, ml/min/1.73 m^2^, mean (SD)	91.6 (16.7)
eGFR stage, n (%)	
≥ 60	7758 (95.9)
45–60	264 (3.3)
30–45	46 (0.5)
<30	25 (0.3)
ACR, mg/g, median (IQR)	6.4 (4.3-11.0)
ACR stage, n (%)	
<30	7419 (91.7)
30–299	570 (7.0)
≥300	103 (1.3)
*Outcomes*	
DKD, n (%) °	271 (29.2)
CKD, n (%)	335 (4.1)

Notes: ABW: adjusted body weight; AGFR: android-to-gynoid fat mass ratio; AGLR: android-to-gynoid lean mass ratio; BMI: body mass index; CKD: chronic kidney disease; DKD: diabetic kidney disease (° prevalence calculated among the 930 patients with diabetes); HDL: high-density lipoprotein; PHDI: Planetary Health Diet Index; TEI: total energy intake.

**Table 3:** Survey-weighted linear regression models showing the associations between continuous PHDI score (each 10-point increase), AGLR, AGFR, and BMI.

PHDI vs. AGLR %	Model A, β (95% CI)	Model B, β (95% CI)
PHDI score	−0.65 (−0.75, −0.54) ***	−0.46 (−0.56, −0.36) ***
Age	0.15 (0.14, 0.17) ***	0.12 (0.10, 0.14) ***
White race	0.78 (0.42, 1.14) ***	1.28 (0.92, 1.64) ***
Female gender	0.69 (0.38, 0.99) ***	1.46 (1.12, 1.80) ***
Diabetes	-	4.41 (3.95, 4.88) ***
Hypertension	-	1.24 (0.91, 1.57) ***
CKD	-	−0.67 (−1.45, 0.11)
Heart disease		1.08 (0.12–2.03) *
Respiratory disease		2.16 (1.48–2.85) ***
Stroke		0.74 (−0.03, 1.52)
Cancer		0.24 (−0.35, 0.82)
HDL	-	−0.07 (−0.08, 0.06) ***
TEI (Kcal/day)	-	
1500–3000	-	Reference
<1500	-	0.11 (−0.26, 0.49)
>3000	-	0.39 (−0.08, 0.86)
**PHDI vs. AGFR %**	**Model A, β (95%CI)**	**Model B, β (95%CI)**
PHDI score	−0.61 (−0.80, −0.51) ***	−0.69 (−0.99,−0.39) ***
Age	0.15 (0.14–0.17) ***	0.14 (0.10–0.19) ***
White race	0.78 (0.42–1.14) ***	1.28 (0.46–2.09) **
Female gender	0.69 (0.38–0.99) ***	−15.38 (−16.47–−14.28) ***
Diabetes	-	8.62 (7.48–9.76) ***
Hypertension	-	4.85 (3.88–5.82) ***
CKD	-	−0.93 (−2.95–1.10)
Heart disease		2.01 (−0.27, 4.28)
Respiratory disease		1.19 (−0.44, 2.83)
Stroke		1.50 (−0.87, 3.87)
Cancer		−0.65 (−2.17, 0.86)
HDL	-	−0.33 (−0.36–−0.30) ***
TEI (Kcal/day)	-	-
1500–3000	-	Reference
<1500	-	0.83 (−0.31–1.97)
>3000	-	0.05 (−1.34–1.44)
**PHDI vs. BMI**	**Model A, β (95%CI)**	**Model B, β (95%CI)**
PHDI score	−0.47 (−0.60, −0.34) ***	−0.26 (−0.38, −0.14) ***
Age	−0.03 (−0.05, −0.01) **	−0.05 (−0.08, −0.03) ***
White race	−0.85 (−1.18, −0.53) ***	−0.28 (−0.61, −0.06) *
Female gender	0.50 (0.13, 0.87) **	1.98 (1.55, 2.41) ***
Diabetes	-	3.00 (2.39, 3.61) ***
Hypertension	-	2.22 (1.86, 2.58) ***
CKD	-	−0.26 (−1.14, 0.62)
Heart disease		0.59 (−0.19, 1.36)
Respiratory disease		0.29 (−0.35, 0.92)
Stroke		−0.59 (−1.93, 0.75)
Cancer		−0.40 (−0.92, 0.11)
HDL	-	−0.11 (−0.13,−0.10) ***
TEI (Kcal/day)	-	-
1500–3000	-	Reference
<1500	-	−0.45 (−0.94, 0.04)
>3000	-	0.08 (−0.33, 0.49)

Notes: AGFR: android-to-gynoid fat mass ratio; AGLR: android-to-gynoid lean mass ratio; BMI: body mass index; CKD: chronic kidney disease; HDL: high-density lipoprotein; PHDI: Planetary Health Diet Index; TEI: total energy intake; *** *p* value < 0.001; ** *p* value < 0.01; * *p* value < 0.05.

**Table 4:** Survey-weighted logistic regression models of the association between continuous PHDI score (each 10-point increase) and CKD in the whole study population.

	Model A, OR (95%CI)	Model B, OR (95%CI)	Model C, OR (95%CI)	Model D, OR (95%CI)
PHDI score	0.87 (0.80–0.95) **	0.91 (0.83–0.99) *	0.91 (0.83–0.99) *	0.91 (0.83–0.99) *
Age	1.12 (1.11–1.14) ***	1.10 (1.09–1.12) ***	1.10 (1.09–1.12) ***	1.10 (1.09–1.12) ***
White race	0.61 (0.48–0.79) ***	0.71 (0.54–0.92) *	0.71 (0.55–0.93) *	0.73 (0.55–0.95) *
Female gender	1.36 (1.03–1.81) *	1.23 (0.83–1.80)	1.20 (0.81–1.80)	1.03 (0.67–1.59)
Diabetes		1.57 (1.11–2.22) **	1.53 (1.08–2.18) **	1.64 (1.14–2.35) **
Hypertension	-	2.12 (1.55–2.89) ***	2.08 (1.51–2.37) ***	2.10 (1.53–2.89) ***
Heart disease		2.09 (1.46–2.99) ***	2.10 (1.45–3.02) ***	2.11 (1.47–3.02) ***
Respiratory disease		1.11 (0.74–1.65)	1.10 (0.74–1.64)	1.10 (0.74–1.65)
Stroke		1.86 (1.09–3.16) *	1.88 (1.09–3.23) *	1.94 (1.12–3.34) *
Cancer		1.32 (0.94–1.87)	1.30 (0.92–1.84)	1.32 (0.93–1.87)
HDL	-	0.99 (0.98–1.00)	0.99 (0.98–1.01)	0.99 (0.98–1.00)
TEI (Kcal/day)				
1500–3000	-	Reference	Reference	Reference
<1500	-	1.68 (1.20–2.35) **	1.70 (1.21–2.39) ***	1.72 (1.22–2.43) ***
>3000	-	0.61 (0.34–1.09)	0.61 (0.34–1.10)	0.60 (0.34–1.08)
Obesity	-	-	1.09 (0.76–1.57)	1.13 (0.78–1.62)
AGLR Q1	-	-	Reference	-
AGLR Q2	-	-	0.89 (0.60–1.31)	-
AGLR Q3	-	-	0.97 (0.66–1.42)	-
AGFR Q1	-	-	-	Reference
AGFR Q2	-	-	-	1.13 (0.82–1.56)
AGFR Q3	-	-	-	0.71 (0.50–1.07)

Notes: AGFR: android-to-gynoid fat mass ratio; AGLR: android-to-gynoid lean mass ratio; HDL: high-density lipoprotein; PHDI: Planetary Health Diet Index; TEI: total energy intake; *** *p* value < 0.001; ** *p* value < 0.01; * *p* value < 0.05.

**Table 5:** Survey-weighted logistic regression models of the association between continuous PHDI (each 10-point increase) and DKD in the subsample of patients with diabetes mellitus.

	Model A, OR (95%CI)	Model B, OR (95%CI)	Model C, OR (95%CI)	Model D, OR (95%CI)
PHDI score	0.85 (0.76–0.96) **	0.85 (0.75–0.97) *	0.87 (0.77–0.99) *	0.86 (0.76–0.97) *
Age	1.06 (1.04–1.08) ***	1.05 (1.03–1.07) ***	1.05 (1.03–1.07) ***	1.05 (1.04–1.07) ***
White race	0.54 (0.38–0.76) ***	0.53 (0.38–0.76) ***	0.46 (0.32–0.656) ***	0.50 (0.35–0.71) ***
Female gender	0.66 (0.43–1.02)	0.60 (0.38–0.94) *	0.52 (0.32–0.84) **	0.65 (0.41–1.05)
Hypertension	-	1.67 (1.20–2.32) ***	1.60 (1.14–2.25) **	1.65 (1.18–2.32) **
Heart disease		2.28 (1.31–3.96) **	2.37 (1.29–4.35) **	2.32 (1.32–4.10) **
Respiratory disease		0.63 (0.38–1.06)	0.61 (0.37–1.01)	0.60 (0.36–1.01)
Stroke		1.76 (0.99–3.10)	1.67 (0.93–2.99)	1.71 (1.00–2.95) *
Cancer		0.95 (0.54–1.66)	0.90 (0.51–1.60)	0.90 (0.50–1.63)
HDL	-	1.00 (0.98–1.01)	1.00 (0.99–1.02)	1.00 (0.98–1.01)
TEI (Kcal/day)				
1500–3000	-	Reference	Reference	Reference
<1500	-	1.57 (1.03–2.39) *	1.56 (1.02–2.40) *	1.57 (1.03–2.40) *
>3000	-	0.82 (0.47–1.45)	0.82 (0.46–1.43)	0.83 (0.47–1.47)
Obesity	-	-	1.03 (0.75–1.42)	1.18 (0.88–1.57)
AGLR Q1	-	-	Reference	-
AGLR Q2	-	-	1.02 (0.56–1.85)	-
AGLR Q3	-	-	2.40 (1.39–4.16) **	-
AGFR Q1			-	Reference
AGFR Q2			-	0.54 (0.29–1.03)
AGFR Q3			-	0.96 (0.53–1.73)

Notes: AGFR: android-to-gynoid fat mass ratio; AGLR: android-to-gynoid lean mass ratio; HDL: high-density lipoprotein; PHDI: Planetary Health Diet Index; TEI: total energy intake; *** *p* value < 0.001; ** *p* value < 0.01; * *p* value < 0.05.

## Data Availability

The data used in this paper is reported at https://wwwn.cdc.gov/nchs/nhanes/default.aspx (accessed on 31 July 2025).

## Supplementary Materials

The following supporting information can be downloaded at: https://www.mdpi.com/article/10.3390/nu17162692/s1; Table S1: Main characteristics of patients belonging to different PHDI score quintiles; Table S2: Survey-weighted logistic regression models of the association between PHDI score quintiles and CKD in the study population; Table S3: Survey-weighted logistic regression models of the association between PHDI score quintiles and DKD in the subsample of patients with diabetes mellitus.

## Abbreviations

The following abbreviations are used in this manuscript:

AGFR	Android-to-gynoid fat mass ratio
AGLR	Android-to-gynoid lean mass ratio
BMI	Body mass index
CKD	Chronic kidney disease
DKD	Diabetic kidney disease
DXA	Dual-energy X-ray absorptiometry
GFR	Glomerular filtration rate
HDL	High-density lipoprotein
NHANES	National Health and Nutrition Examination Survey
PHD	Planetary Health Diet
PHDI	Planetary Health Diet Index
TEI	Total energy intake
TNF	Tumor necrosis factor
USDA	United States Department of Agriculture
