# Supplementary material for: Planetary Health Diet and Body Mass Distribution in Relation to Kidney Health: Evidence from NHANES 2003–2018
Source: Nutrients. 2025 Aug 20;17(16):2692. doi: 10.3390/nu17162692 (PMC12389508; doi:10.3390/nu17162692)
Supplement: Supplementary file 1 [file nutrients-17-02692-s001.zip › nutrients-3775660-supplementary.pdf]

Supplementary Table S1. Main characteristics of patients belonging to different PHDI score quintiles

	Q1 (21.2-60.1)	Q2 (60.2-69.7)	Q3 (69.8-77.6)	Q4 (77.7-86.0)	Q5 (86.1-127.8)	p value
**N**						
**Age, mean (SD)**	50.5 (7.2)	51.7 (7.9)	52.9 (8.7)	52.9 (8.9)	52.9 (9.4)	<0.001
**Female gender, n (%)**	619 (38.2)	765 (47.3)	921 (57.0)	915 (56.5)	989 (61.0)	<0.001
**White race, n (%)**	1,192 (73.6)	1,151 (71.1)	1,169 (72.3)	1,191 (73.6)	1,175 (72.5)	0.595
**Hypertension, n (%)**	613 (38.0)	641 (39.6)	584 (36.2)	565 (34.9)	472 (29.2)	<0.001
**Glycated hemoglobin, mean (SD)**	5.7 (1.0)	5.7 (1.0)	5.7 (1.0)	5.7 (1.0)	5.6 (0.8)	0.001
**Diabetes, n (%)**	184 (11.4)	196 (12.1)	183 (11.3)	206 (12.7)	161 (10.0)	0.308
**Heart disease, n (%)**	74 (4.5)	81 (5.0)	70 (4.6)	57 (3.5)	52 (3.1)	0.161
**Respiratory disease, n (%)**	167 (10.3)	142 (8.8)	121 (7.5)	118 (7.0)	86 (5.3)	0.003
**Stroke, n (%)**	52 (3.2)	38 (2.4)	39 (2.4)	28 (1.7)	20 (1.3)	0.013
**Cancer, n (%)**	128 (8.0)	140 (8.7)	159 (9.8)	163 (10.0)	145 (8.9)	0.585
**AGLR, median (IQR)**	0.53 (0.49-0.57)	0.53 (0.49-0.56)	0.52 (0.48-0.55)	0.52 (0.48-0.55)	0.50 (0.48-0.54)	<0.001
**AGFR, median (IQR)**	0.61 (0.48-0.73)	0.59 (0.47-0.73)	0.54 (0.43-0.68)	0.54 (0.43-0.68)	0.50 (0.38-0.64)	<0.001
**BMI, mean (SD)**	29.7 (6.4)	30.0 (6.3)	29.3 (6.2)	29.1 (5.9)	27.8 (2.2)	<0.001
**Obesity, n (%)**	707 (44.0)	713 (44.1)	625 (38.8)	611 (37.7)	456 (28.2)	<0.001
**HDL, mg/dl, median (IQR)**	50.5 (15.4)	52.8 (16.7)	55.1 (17.7)	55.4 (16.6)	58.3 (17.4)	<0.001
**Total daily intake, Kcal/day, median (IQR)**	2,167 (1,659-2,710)	2,070 (1,575-2,593)	1,893 (1,458-2,471)	1,927 (1,499-2,533)	1,907 (1,497-2,382)	<0.001
**1500-3000**	1,046 (64.7)	1,045 (64.6)	1,010 (62.4)	999 (61.7)	1,080 (66.7)	<0.001
**<1500**	300 (18.5)	338 (20.8)	429 (26.5)	405 (25.0)	406 (25.1)	
**>3000**	271 (16.7)	236 (14.6)	178 (11.0)	216 (13.3)	133 (82.)	
**Total protein/ABW (g/Kg/day), median (IQR)**	1.2 (0.9-1.5)	1.2 (0.9-1.5)	1.2 (0.9-1.4)	1.1 (0.9-1.4)	1.1 (0.9-1.4)	0.001
**eGFR, ml/min/1.73 m², median (IQR)**	94.3 (82.8-106.0)	94.3 (82.3-105.3)	92.8 (79.0-104.0)	94.1 (80.7-105.3)	93.1 (80.5-104.7)	0.037
**ACR, mg/g, median (IQR)**	6.3 (4.3-10.7)	6.6 (4.4-11.9)	6.2 (4.4-11.4)	6.3 (4.1-10.6)	6.6 (4.4-10.3)	0.301
Study outcomes						
**DKD, n (%)°**	54 (29.2)	59 (30.0)	67 (36.7)	61 (29.8)	30 (18.6)	0.043
**CKD, n (%)**	53 (3.3)	65 (4.0)	104 (6.4)	67 (4.1)	47 (2.9)	<0.001

Notes: ABW: adjusted body weight; AGFR: android-to-gynoid fat mass ratio; AGLR: android-to-gynoid lean mass ratio; BMI: body mass index; CKD: chronic kidney disease; DKD: diabetic kidney disease (° prevalence calculated among the 930 patients with diabetes); HDL: high density lipoprotein; PHDI: planetary health diet index; TEI: total energy intake.

Supplementary Table S2. Survey-weighted logistic regression models of the association between PHDI score quintiles and CKD in the study population

	Model A, OR (95%CI)	Model B, OR (95%CI)	Model C, OR (95%CI)	Model D, OR (95%CI)
**PHDI score Q1**	**Reference**	**Reference**	**Reference**	**Reference**
**PHDI score Q2**	0.97 (0.61–1.55)	1.08 (0.67–1.75)	1.08 (0.67–1.76)	1.07 (0.66–1.73)
**PHDI score Q3**	1.31 (0.84–2.03)	1.50 (0.95–2.39)	1.50 (0.93–2.41)	1.47 (0.92–2.34)
**PHDI score Q4**	0.76 (0.49–1.18)	0.87 (0.54–1.41)	0.88 (0.55–1.40)	0.87 (0.54–1.39)
**PHDI score Q5**	0.56 (0.36–0.88)*	0.70 (0.43–1.14)	0.70 (0.43–1.15)	0.69 (0.43–1.12)
Age	1.12 (1.11–1.14)***	1.10 (1.09–1.12)***	1.10 (1.09–1.12)***	1.10 (1.09–1.12)***
White race	0.62 (0.48–0.79)***	0.71 (0.54–0.92)*	0.71 (0.55–0.93)*	0.72 (0.55–0.95)*
Female gender	1.32 (0.99–1.76)	1.21 (0.81–1.79)	1.19 (0.79–1.78)	1.03 (0.67–1.59)
Diabetes	-	1.56 (1.10–2.21)*	1.53 (1.08–2.17)*	1.63 (1.14–2.32)**
Hypertension	-	2.12 (1.55–2.90)***	2.09 (1.52–2.89)***	2.11 (1.53–2.90)***
Heart disease	-	2.11 (1.47–3.03)***	2.13 (1.47–3.07)***	2.14 (1.49–3.06)***
Respiratory disease	-	1.14 (0.76–1.71)	1.13 (0.75–1.69)	1.13 (0.75–1.70)
Stroke	-	1.86 (1.09–3.18)*	1.88 (1.09–3.24)*	1.94 (1.12–3.35)*
Cancer	-	1.31 (0.93–1.85)	1.29 (0.91–1.83)	1.31 (0.93–1.86)
HDL	-	0.99 (0.98–1.00)	0.99 (0.98–1.01)	0.99 (0.98–1.00)
TEI category (Kcal/day)				
1500-3000	-	Reference	Reference	Reference
<1500	-	1.65 (1.18–2.29)**	1.67 (1.20–2.34)**	1.69 (1.21–2.36)**
>3000	-	0.63 (0.35–1.12)	0.63 (0.35–1.13)	0.62 (0.34–1.11)
Obesity	-	-	1.07 (0.75–1.54)	1.10 (0.77–1.58)
AGLR tertiles				
Q1	-	-	Reference	-
Q2	-	-	0.88 (0.59–1.30)	-
Q3	-	-	0.96 (0.65–1.42)	-
AGFR tertiles				-
Q1	-	-	-	Reference
Q2	-	-	-	1.15 (0.83–1.59)
Q3	-	-	-	0.76 (0.52–1.11)

Notes: AGFR: android-to-gynoid fat mass ratio; AGLR: android-to-gynoid lean mass ratio; BMI: body mass index; CKD: chronic kidney disease; DKD: diabetic kidney disease; HDL: high density lipoprotein; PHDI: planetary health diet index; TEI: total energy intake; *** p value < 0.001; ** p value < 0.01; * p value <0.01.

Supplementary Table S3. Survey-weighted logistic regression models of the association between PHDI score quintiles and DKD in the subsample of patients with diabetes mellitus

	**Model A, OR (95%CI)**	**Model B, OR (95%CI)**	**Model C, OR (95%CI)**	**Model D, OR (95%CI)**
**PHDI score Q1**	**Reference**	**Reference**	**Reference**	**Reference**
**PHDI score Q2**	0.87 (0.46–1.62)	0.79 (0.45–1.37)	0.76 (0.44–1.33)	0.76 (0.43–1.35)
**PHDI score Q3**	1.19 (0.69–2.06)	1.18 (0.65–2.14)	1.21 (0.67–2.19)	1.19 (0.65–2.19)
**PHDI score Q4**	0.65 (0.35–1.17)	0.77 (0.44–1.34)	0.78 (0.45–1.35)	0.77 (0.44–1.36)
**PHDI score Q5**	0.45 (0.24–0.83)*	0.47 (0.26–0.85)*	0.51 (0.28–0.93)*	0.46 (0.26–0.82)**
**Age**	1.06 (1.04–1.08)***	1.05 (1.03–1.06)***	1.05 (1.03–1.07)***	1.05 (1.03–1.07)***
**White race**	0.48 (0.33–0.72)***	0.53 (0.37–0.75)***	0.45 (0.32–0.65)***	0.49 (0.34–0.71)***
**Female gender**	0.64 (0.42–0.97)*	0.58 (0.37–0.91)*	0.51 (0.31–0.81)**	0.65 (0.40–1.04)
Hypertension	-	1.65 (1.19–2.29)**	1.59 (1.13–2.23)**	1.63 (1.17–2.29)**
Heart disease	-	2.39 (1.39–4.10)**	2.52 (1.39–4.55)**	2.46 (1.41–4.27)**
Respiratory disease	-	0.64 (0.38–1.08)	0.62 (0.37–1.03)	0.61 (0.36–1.04)
Stroke	-	1.70 (0.96–3.01)	1.60 (0.88–2.91)	1.66 (0.96–2.88)
Cancer	-	0.94 (0.53–1.68)	0.90 (0.50–1.62)	0.89 (0.49–1.65)
HDL	-	1.00 (0.98–1.01)	1.00 (0.99–1.02)	1.00 (0.98–1.01)
TEI category (Kcal/day)				
1500-3000	-	Reference	Reference	Reference
<1500	-	1.54 (1.03–2.32)*	1.54 (1.01–2.34)*	1.55 (1.03–2.33)*
>3000	-	0.84 (0.47–1.50)	0.84 (0.47–1.50)	0.85 (0.47–1.52)
Obesity	-	-	1.01 (0.73–1.40)	1.08 (0.80–1.45)
AGLR Q1	-	-	Reference	-
AGLR Q2	-	-	1.04 (0.57–1.90)	-
AGLR Q3	-	-	2.46 (1.41–4.31)**	-
AGFR Q1			-	**Reference**
AGFR Q2			-	0.54 (0.29–1.02)
AGFR Q3			-	1.01 (0.56–1.82)

Notes: AGFR: android-to-gynoid fat mass ratio; AGLR: android-to-gynoid lean mass ratio; BMI: body mass index; CKD: chronic kidney disease; DKD: diabetic kidney disease; HDL: high density lipoprotein; PHDI: planetary health diet index; TEI: total energy intake; *** p value < 0.001; ** p value < 0.01; * p value <0.01.
